# Immortalization of human adipose-derived stromal cells: production of cell lines with high growth rate, mesenchymal marker expression and capability to secrete high levels of angiogenic factors

**DOI:** 10.1186/scrt452

**Published:** 2014-05-06

**Authors:** Luigi Balducci, Antonella Blasi, Marilisa Saldarelli, Antonio Soleti, Augusto Pessina, Arianna Bonomi, Valentina Coccè, Marta Dossena, Valentina Tosetti, Valentina Ceserani, Stefania Elena Navone, Maria Laura Falchetti, Eugenio Agostino Parati, Giulio Alessandri

**Affiliations:** 1 Medestea Research and Production Laboratories, Consorzio CARSO, Bari, Italy; 2 Department of Biomedical, Surgical and Dental Sciences, University of Milan, Milan, Italy; 3 Department of Cerebrovascular Diseases, Fondazione IRCCS Neurological Institute Carlo Besta, Milan, Italy; 4 Laboratory of Experimental Neurosurgery and Cell Therapy, Neurosurgery Unit, Fondazione IRCCS Ca’Granda Ospedale Maggiore Policlinico, Milan, Italy; 5 Institute of Cell Biology and Neurobiology, CNR, Rome, Italy

## Abstract

**Introduction:**

Human adipose-derived stromal cells (hASCs), due to their relative feasibility of isolation and ability to secrete large amounts of angiogenic factors, are being evaluated for regenerative medicine. However, their limited culture life span may represent an obstacle for both preclinical investigation and therapeutic use. To overcome this problem, hASCs immortalization was performed in order to obtain cells with *in vitro* prolonged life span but still maintain their mesenchymal marker expression and ability to secrete angiogenic factors.

**Methods:**

hASCs were transduced with the human telomerase reverse transcriptase (*hTERT*) gene alone or in combination with either *SV-40* or *HPV E6/E7* genes. Mesenchymal marker expression on immortalized hASCs lines was confirmed by flow cytometry (FC), differentiation potential was evaluated by immunocytochemistry and ELISA kits were used for evaluation of angiogenic factors. Green fluorescent protein (*GFP*) gene transduction was used to obtain fluorescent cells.

**Results:**

We found that *hTERT* alone failed to immortalize hASCs (hASCs-T), while *hTERT/SV40* (hASCs-TS) or *hTERT/HPV E6/E7* (hASCs-TE) co-transductions successfully immortalized cells. Both hASCs-TS and hASCs-TE were cultured for up to one year with a population doubling level (PDL) up to 100. Comparative studies between parental not transduced (hASCs-M) and immortalized cell lines showed that both hASCs-TS and hASCs-TE maintained a mesenchymal phenotypic profile, whereas differentiation properties were reduced particularly in hASCs-TS. Interestingly, hASCs-TS and hASCs-TE showed a capability to secrete significant amount of HGF and VEGF. Furthermore, hASCs-TS and hASCs-TE did not show tumorigenic properties *in vitr*o although some chromosomal aberrations were detected. Finally, hASCs-TS and hASCs-TE lines were stably fluorescent upon transduction with the *GFP* gene.

**Conclusions:**

Here we demonstrated, for the first time, that hASCs, upon immortalization, maintain a strong capacity to secrete potent angiogenic molecules. By combining hASCs immortalization and their paracrine characteristics, we have developed a “hybridoma-like model” of hASCs that could have potential applications for discovering and producing molecules to use in regenerative medicine (process scale-up).

In addition, due to the versatility of these fluorescent-immortalized cells, they could be employed in *in vivo* cell-tracking experiments, expanding their potential use in laboratory practice.

## Introduction

Human adipose stromal cells (hASCs) have various practical advantages compared to mesenchymal stromal cells (MSCs) isolated from other tissue sources, such as their ease of being obtained, greater stem cell yields than from other stem cell reservoirs and, most importantly, minimal invasive procedures. These practical aspects make hASCs a real and powerful therapeutic tool for the treatment of numerous human diseases [1,2]. However, to date, translation of MSCs’ preclinical results to the bedside still have serious problems to be solved. One of them certainly relates to the high variability of MSC preparations among different laboratories. The reasons for the variability are multiple and can include the tissue origin of the MSCs (fat, bone marrow, umbilical cord blood and so on), the gender and age of the donors, as well as the methods of isolation and the culture conditions used [3-5]. Besides this, the use of MSCs in clinical care is also limited by technical problems regarding their particularly limited life-span for *ex-vivo* expansion [6]. In general, MSCs can easily adapt to culture conditions and, particularly in the early stages of culture, they show a good proliferative rate. But, during their expansion, whatever their tissue origin, and the age or gender of the donor, MSCs undergo senescence and significantly decrease cell growth sometime after a very limited number of *in vitro* cell passages [7,8]. This growth limit definitely represents a serious problem related to both MSCs and hASCs, because usually a significant number of cells and multiple cell treatments might be required for treating human diseases.

A possible solution to circumvent MSCs’ preparation heterogeneity and their limited growth expansion is immortalization by genetic manipulation. Generally, this strategy requires abrogation of p53 and pRB-mediated terminal proliferation and/or activation of a telomerase reverse transcriptase (*hTERT*) maintenance mechanism [9]. Several methods have been developed for immortalizing cells *in vitro*[10]. Among these, the introduction of viral genes, such as *SV40*[11] or human papilloma-virus (HPV) *E6/E7* genes [12] and the *hTERT* gene [13-15] have been widely used. On this basis, the aim of the present work was to immortalize different hASC preparations in order: 1) to produce new human stromal cell lines with more stable characteristics to be used both *in vitro* and *in vivo* in preclinical investigations, and 2) to use these cell lines as a source for the isolation and production of angiogenic factors.

Here we show that by combining *hTERT* with either *SV40* or *E6/E7*, we were able to produce eight different hASC cell lines. All cell lines were characterized by flow cytometry and expanded *in vitro* up to 100 population doubling levels (PDL). The cells maintained their typical mesenchymal marker expression and an elevated capability to secrete angiogenic factors, such as hepatocyte growth factor (HGF) and vascular endothelial growth factor (VEGF), in the culture medium. We conclude that hASCs are ideal to produce immortalized hMSC cell lines that are able to maintain their phenotype and their functional characteristics. These cells could be exploited for the identification and extraction of hASCs-derived angiogenic molecules that could be used in regenerative medicine. Finally, by coupling hASCs immortalization and their paracrine characteristics, we have developed a “hybridoma-like model” that may have a potential application in discovering and producing molecules to use in regenerative medicine (process scale-up).

## Methods

### Isolation of hASCs

After approval by the Ethical Committee of “F. Miulli” Hospital (Acquaviva, Bari, Italy), human fat specimens were obtained from four patients undergoing abdominal surgery. Informed consent was obtained from all patients in this study. Isolation of cells was performed as previously described [16] and four different hASCs cell populations were generated and subsequently used to be immortalized. All hASCs cells were cultured in a EGM Bullet kit (Lonza, Verviers, Belgium) supplemented with 10% FCS, antibiotics and L-Glutamine 2 mM, at 37°C, 5% CO_2_. *In vitro* cell growth was monitored by cell number count and cumulative PDL calculation. Population doubling (PD) gained at each passage was determined using the following formula: PD_[n/(n-1)]_ = (Log(Nn / Nn-1))/Log 2, where n: passage, n-1: previous passage, Nn: cell number at passage n and Nn-1: cells plated at passage n-1. Cumulative PDL is the sum of PDs [17].

### Lentiviral transduction of hASCs

Viral transduction of hASCs is a well-established method to transduce genes into cells and obtain their expression [18]. To induce hASCs immortalization, hTERT alone, or in combination with *SV40* and *E6/E7* genes, was used. The Lentiviral vectors pLenti-*hTERT*, pLenti-III-*SV40* and pLenti-III-HPV-16 *E6/E7* were purchased from Applied Biological Materials Inc. (Richmond, BC, Canada). Cells were transduced according to the manufacturer’s protocol. Briefly, 5.0 × 10^3^ cells/cm^2^ were plated in six-well tissue culture plates and incubated at 37°C, 5% CO_2_ until they reached 65 to 70% confluence. Afterwards, medium was replenished with pLenti-*hTERT* viral suspension and fresh medium (ratio 1:1) in the presence of 8 μg/ml polybrene. Cells were then incubated overnight at 37°C, 5% CO_2_. After 24 hours, the medium was changed and cells were left in culture for three days and then detached with trypsin. An identical scheme was followed for pLenti-III-*SV40* and pLenti-III-HPV-16 *E6/E7* lentiviral transduction. For each hASC-T population, an aliquot of cells was transduced with either the *SV40* or *E6/E7* gene. All cells were grown in the above-mentioned medium and the following nomenclature was assigned: M, non-transduced (Mother, putative cell preparations); T, *hTERT*-transduced; TS, *hTERT/SV40*-transduced; TE, *hTERT/E6/E7*-transduced.

### Doubling time assay

To estimate the time required by cells to duplicate their number, doubling time assays were performed. A total of 13.0 × 10^3^ cells/cm^2^ were plated in 12-well tissue culture plates (in triplicate) with 1 ml/well of culture medium and incubated at 37°C, 5% CO_2_, for 24, 48, 72 and 96 hours. At each time point, cells were washed once with 2 ml/well PBS 1X, detached with 0.5 ml/well trypsin/EDTA, resuspended in 1.5 ml/sample in complete culture medium and counted in a hemacytometer. We obtained three different cell count values for all samples tested (M, T, TS and TE, respectively).

### Cell morphology and senescence-associated β-galactosidase assay

Cell morphology of hASCs-M, hASCs-T, hASCs-TS and hASCs-TE was observed and analyzed using a phase contrast microscope (CKX41, Olympus, Tokyo, Japan). Cells were than photographed at 10X magnification with a C-7070 digital compact Camera (Olympus,).

Senescence-associated β-galactosidase activity was evaluated with a senescence cell histochemical staining kit (SIGMA Aldrich, USA), according to protocol instructions. Briefly, 3.0 × 10^3^ cells/cm^2^ were plated in 12-well tissue culture plates (in triplicate) and incubated at 37°C, 5% CO_2_ for 48 hours. Cells were then washed twice with 1 ml/well PBS 1X and fixed for seven minutes at room temperature. After three washes, cells were stained at 37°C overnight and visualized under a microscope.

### Immunophenotypic characterization

Cultures of hASCs-M, hASCs-T, hASCs-TS and hASCs-TE were phenotypically characterized by flow cytometry (FC). After trypsinization, cells were resuspended with FC buffer (pH 7.2 PBS, BSA 0.5%, sodium azide 0.02%) at a concentration of 0.1 x 10^6^/100 μl. Cells were incubated with fluorescently labeled antibodies for 30 minutes at room temperature in a dark room and then washed with FC buffer to remove non-conjugated antibodies. Fluorescein isothiocyanate (FITC-F) or phycoerythrin (PE) conjugate-antibodies were used: CD90PE, CD105PE, CD34PE, CD45F, CD44F, CD106PE, CD146PE (Immunotech®, Milan, Italy), HLA-IF, HLA-IIPE (Biolegend®, Italy), CD73PE, SSEA-4PE (R&D®, Milan, Italy). Epics "XL-MCL" (Beckman Coulter, USA) flow cytometer was used for analyzing fluorescent immunophenotypic marker signals. At least 10,000 events for test samples were acquired. Sample histogram elaboration was performed with EXPO 32 software to assess fluorescent distribution.

### Osteogenic and adipogenic differentiation

hASCs differentiation capability was assessed by investigating osteogenic and adipogenic induction. For osteogenic differentiation, cells were seeded into six-well culture plates and at a density of 5.0 × 10^3^ cells/cm^2^ and cultivated in NH OsteoDiff medium (Miltenyi Biotec, GmBH) for two weeks. Osteogenic potential was assessed by alkaline phosphatase activity with SIGMA FAST BCIP/NBT substrate (SIGMA-Aldrich, USA).

For adipogenic differentiation, cells were placed into six-well culture plates at a density of 5.0 × 10^3^ cells/cm^2^ and cultivated in NH AdipoDiff medium (Miltenyi Biotec, GmBH) for three weeks. Adipogenic potential was assessed by Oil Red O (SIGMA-Aldrich, USA) staining.

### HGF AND VEGF secretion

To evaluate HGF and VEGF secreted by hASCs-M, hASCs-T, hASCs-TS and hASCs-TE cultured cells, cell supernatants were collected and analyzed as follows. A total of 20.0 × 10^4^ cells/cm^2^ were plated in T-25 flasks with 4 ml complete medium at 37°C, 5% CO_2_ for 72 hours. At the end of incubation, cell supernatants were harvested and centrifuged at 2,500 rpm for 10 minutes at 4°C to remove cell debris, and cryopreserved at −80°C until use. Growth factor production was tested by enzyme immunoassay ELISA kits (Human VEGF and HGF Quantikine ELISA Kit, R&D Systems, Minneapolis, MN, USA). Protein concentration was measured following the standard guidelines illustrated in the ELISA kit used. The culture medium alone (containing FBS) was used as a negative control. Absorbance was measured with a microplate photometric reader DV990BV4 (GDV, Italy). Cytokine concentration in conditioned media was obtained after subtracting the value obtained by the negative control (background value). For each sample analyzed, HGF and VEGF secretion were normalized by the total number of cells and expressed as pg/10^5^ cells.

### Karyotyping

Mid-log cell cultures were arrested with 100 ng/ml colcemid, disrupted in 0.075 M KCl solution at 37°C, fixed with a 3:1 mixture of methanol and acetic acid, and allowed to air dry. Following brief trypsinization, chromosomes were stained with quinacrine dihydrocloride (SIGMA, St. Louis, MO, USA). For all cell lines analyzed (hASC-M, hASC-T, hASC-TS and hASC-TE) karyotyping was carried out at the following passages: 10, 16, 19 and 26. Karyogram analyses were performed using Leica CW4000 Karyo V1.1 software (Leica Imaging Systems, Cambridge, UK).

### Cellular transformation

To detect *in vitro* cellular transformation, a soft-agar assay was performed. Briefly, 12-well tissue culture plates were coated with 1:1 mixture of complete medium and 1% base agar (SIGMA-Aldrich, USA) and, after solidification, cells were seeded in triplicate at a density of 2.5 × 10^3^ cells/cm^2^. Cells were plated in a 1:1 mixture of complete medium and 0.7% agarose (Lonza, Rockland, ME USA), fed three times a week and incubated at 37°C, 5% CO_2_ for 28 days. Cell colonies were fixed with 1% formaldehyde, stained with 0.05% Crystal Violet, and counted. PC-3 cells (prostate tumor) were used as a positive control.

### Green fluorescent protein (GFP)-transduction

To obtain hASCs-TS and hASCsTE-fluorescent cells, two different approaches were used. Using the first method, as described by Dull *et al*. [19], HEK 293 T cells were transfected with pCCLsin.PPT.hPGK.GFPpre, pMDL, pRSV-REV and pVSV-G plasmids in the presence of Lipofectamine (Invitrogen, Carlsbad, CA, USA). After 48 hours, HEK 293 T cell conditioned medium was filtered and used to transduce hASCs cells. To increase transduction efficiency, polybrene 8 μg/ml was added. hASCs underwent three consecutive infection cycles.

With the second method, lentiviral particles purchased from Lentigen (Baltimore, MD, USA) were used to transduce hASCs according to the manufacturer’s protocol. Both methods generated hASCS that stably expressed GFP and fluorescence did not diminish during the whole period of culture.

### Statistical analysis

Statistical differences were evaluated using Student’s *t*-test where applicable. *P-*values smaller than 0.05 were accepted as significant.

## Results

### Proliferation of hASCs before and after transfection with *hTERT* alone or combined with either *SV40* or *E6/E7*

**Figure 1:**
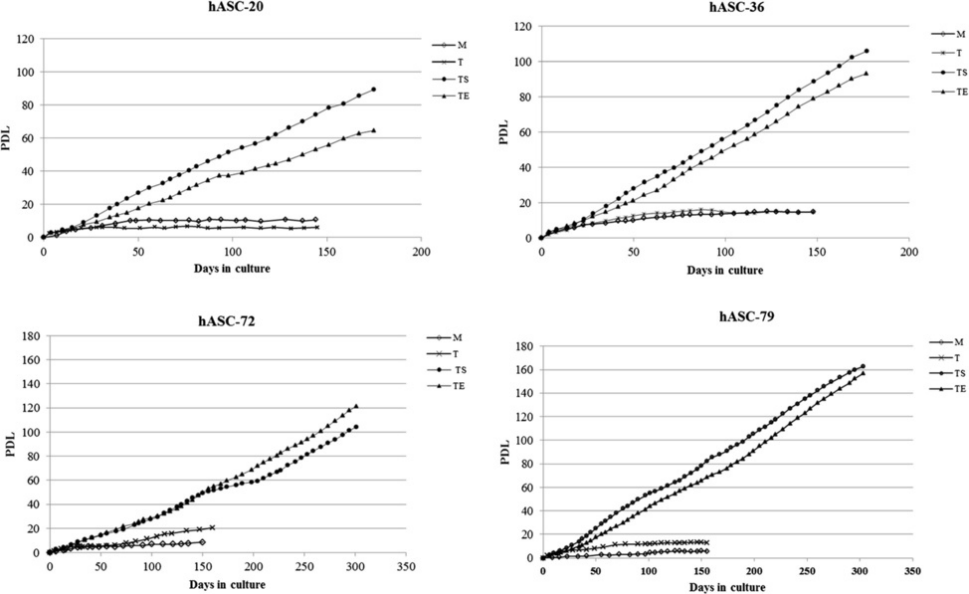
**Growth rate graphics of not transduced (M), hTERT-transduced (T), hTERT/SV40- (TS) and hTERT/E6E7- (TE) co-transduced cells.** Four different hASCs cell lines were tested for cell immortalization, and eight immortalized cell lines were generated (TS or TE). Population Doubling Levels (PDL) were calculated for each cell line over time. PDL represents the sum of PD values obtained when cells were detached and re-plated (see Material and methods for details).

Human fat specimens were obtained from four patients (two males and two females aged between 21 and 59 years) and derived hASC primary cultures named hASC 20, hASC 36, hASC 72 and hASC 79 underwent immortalization procedures. The proliferative rates of all different cultures were followed over time by evaluating the PDL. In Figure 1, the PDL values of the non-transduced original hASCs (−M), *hTERT* transduced (hASCs-T), *hTERT* + *SV40* transduced (hASCs-TS) and hTERT + E6/E7 transduced cells (hASCs-TE) are shown. All non-transduced hASCs (hASC 20-M, hASC 36-M, hASC 72-M and hASC 79-M) showed very low PDL values; they grew slowly in the first 50 days of culture, afterwards they stopped proliferation. All the different hASC-Ms were able to remain in culture for a long time but did not increase their cell number. Transduction of cells with *hTERT* alone slightly increased the PDL values (≤20), with the only exception being hASC-20 T. However, like the non-transfected counterpart, after 50 to 80 days of culture, the cells ceased to proliferate. In contrast, all the hASCs transfected either with the combination *hTERT* + *SV40* (hASCs-TS) or with *hTERT* + *E6/E7* (hASCs-TE) acquired a significant proliferative capability, reaching very high PDL values and still growing after 180 (hASC 20-TS/TE and hASC 36-TS/TE) and 300 (hASC 72-TS/TE and hASC 79-TS/TE) days of culture. Comparing the PDL values of hASCs-TS versus hASCs-TE, we observed that three out of four hASCs-TS (hASC 20-TS, hASC 36-TS and hASC 79-TS) showed a better proliferative activity, suggesting that the *hTERT* + *SV40* co-transduction of hASCs was more efficient than *hTERT* + *E6/E7* in improving growth rate. A summary of the PDL values obtained after 180 and 300 days of hASC-TS and hASC-TE culture is reported in Table 1.

**Table 1:** Days in culture and PDL values of immortalized cells

**Cell line**	**Number of passages before cryo-storing**	**Days in culture**	**PDL VALUE**
hASC-20 TS	45	180	89.48
hASC-20 TE	45	180	64.61
hASC-36 TS	40	180	106.05
hASC-36 TE	40	180	93.40
hASC-72 TS	65	300	104.39
hASC-72 TE	65	300	121.45
hASC-79 TS	65	300	162.48
hASC-79 TE	65	300	156.65

Numerical data of immortalized hASCs. Culture passages prior to cryo-preservation, days in culture and population doubling level (PDL).

**Figure 2:**
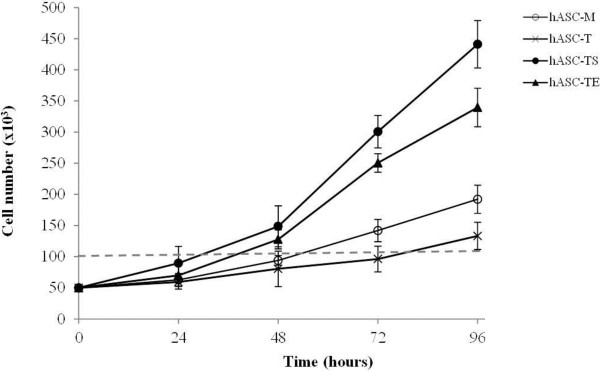
**Doubling time assay of hASC-M, hASC-T, hASC-TS and hASC-TE cells.** A total of 13.0 x 10^6^ cells/cm^2^ were plated at T0 and detached at the indicated time points. Three different values were obtained for each cell line. Cells were evaluated at the following cell culture passages: hASCs-M P7; hASCs-T P17; hASCs-TS and hASCs-TE P21. The histogram represents the mean value ± SD of four different experiments.

To better characterize the effect of different types of transductions on the proliferation of hASCs cultures, we also evaluated the cells doubling time (DT) before and after transfections. As shown in Figure 2, hASC-TS and hASC-TE cells required less time to duplicate when compared to their non-transduced counterpart and hTERT-transduced cells. As reported in Tables 2, the DT mean value was 32.1 ± 8.5 hours for the hASCs-TS at cell passage 21, 39.1 ± 4.2 hours for the hASCs-TE at cell passage 21, 73.8 ± 12.7 hours for the hASCs-T at cell passage 17 and 53.2 ± 9.3 hours for the hASCs-M at cell passage 7. Notably, although hASCs-TS and hASCs-TE were tested at higher cell passages than hASCs-T, the DT mean values of the hASCs-TS and hASCs-TE were much lower than hASCs-T and hASCs-M, thus confirming that subsequent transduction of hASCs-T with SV40 or E6/E7 produced cell lines with very high PDL values.

**Table 2:** Mean doubling time of hASCs cells

***Cell line***	***Mean doubling time (hrs)***	***P-value vs M***	***P-value vs T***
hASC-M	53.2 ± 9.3		
hASC-T	73.8 ± 12.7	0.04	
hASC-TS	32.1 ± 8.5	0.02	0.0004
hASC-TE	39.1 ± 4.2	0.03	0.0006

Mean doubling time values of non-transduced and T-, TS- or TE-transduced cells. Statistical significance vs non-transduced (M) or T-transduced cells.

### Immortalized hASC-TS and hASC-TE cell lines do not display an increase in cell size and β-galactosidase (β-Gal) activity as senescence markers

**Figure 3:**
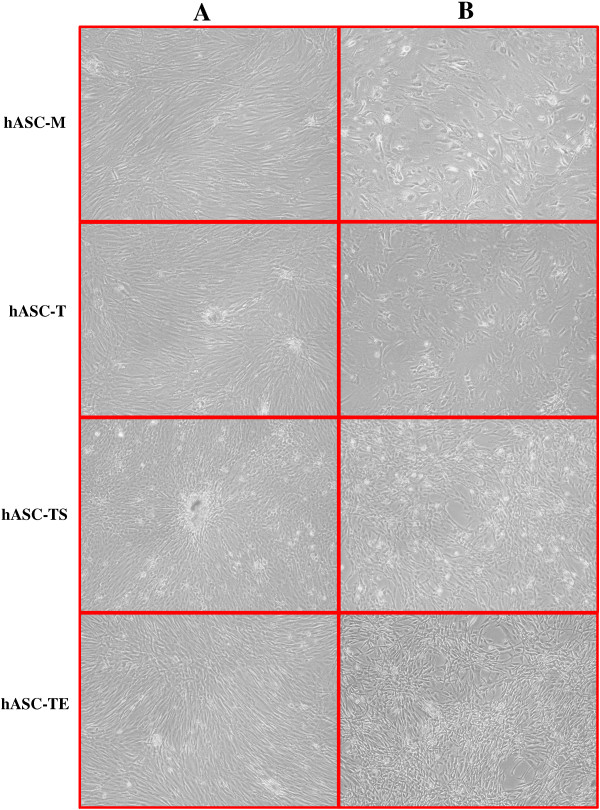
**Cell morphology of hASC-M, −T, −TS and -TE cells. A**: Cells at initial culture period (32 days); **B**: Cells at late culture period (147 days). Magnification 4X.

**Figure 4:**
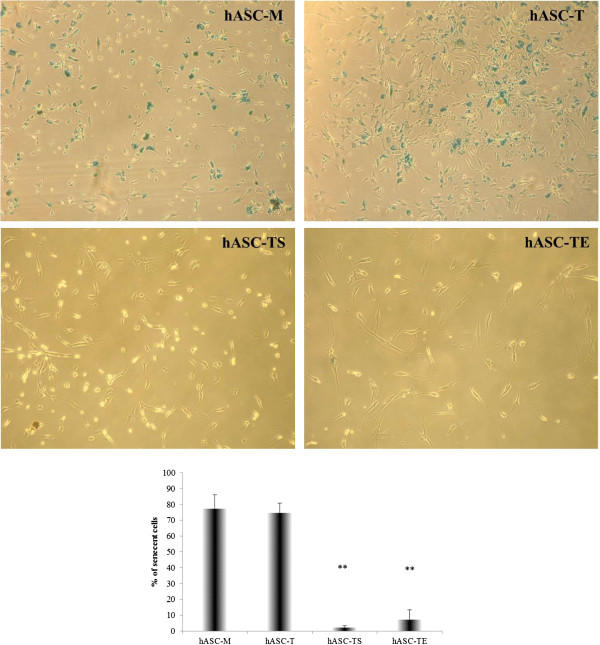
**β-galactosidase (β-gal) senescence evaluation of hASC-M, −T, −TS and -TE cells.** Representative β-gal staining of cells. The percentage of senescent cells were calculated by analyzing eight different fields for each cell line. The histogram represents the mean value ± SD of four different experiments **P <0.01 vs hASCs-M. Magnification 10X.

The change in cell morphology, as well as the expression of β-gal, a marker of senescence, was monitored in order to further characterize the parental and transduced cell lines. Senescent cells usually display an increase of cell size and β-galactosidase activity, that is absent in quiescent, immortal or tumor cells [20]. The morphological changes of different cell lines were analyzed by light microscopy during cell expansion. At an early phase of 32 days of culture, both non- and transduced hASCs showed a similar “fibroblast-like” shape and usually formed an almost uniform cell monolayer at confluence (Figure 3A). After 147 days of culture, hASCs-M and the hASCs-T significantly changed their initial morphology, increasing in cell size and reducing the capability to reach a confluent monolayer, whereas hASCs-TS and hASCs-TE maintained the initial morphology, continuing to generate a very dense cell monolayer even with cell overgrowth in some areas (Figure 3B). Thus, the morphological appearance seems to indicate that only the cells of hASCs-M and hASCs-T underwent a senescence process. To confirm and quantify the senescent cell process, β-gal staining was performed on hASC-M, hASC-T, hASC-TS and hASC-TE cultures. Cells cultured for five months were seeded at low density in order to better detect the positive staining to β-gal. As shown in Figure 4, hASCs-M and hASCs-T showed a significant positivity to β-gal. The calculated percentages of senescent cells were, respectively, 77.21 ± 8.88 and 74.51 ± 6.41. To the contrary, very few positive hASCs-TS and hASCs-TE were detected and the percentages of senescent cells were 2.14 ± 1.11 and 7.15 ± 6.33, respectively (*P* <0.01 vs hASCs-M).

### Immortalized hASC-TS and hASC-TE cell lines express mesenchymal markers, acquire stem cell markers and increase mobilization associated markers

**Figure 5:**
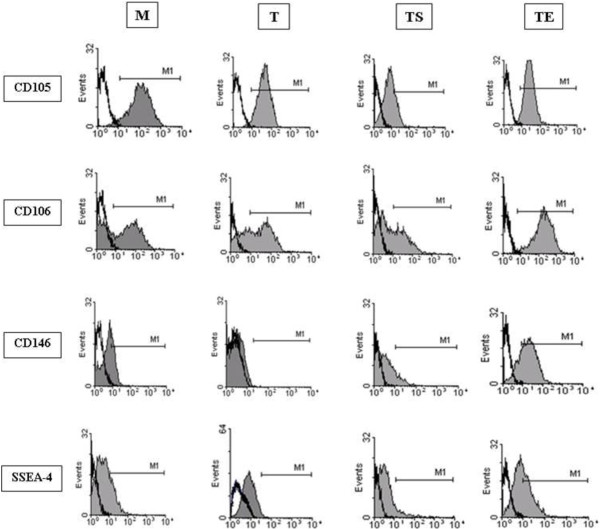
**Flow cytometry histograms of CD105, CD106, CD146 and SSEA-4 surface antigens.** The Mean Fluorescence Intensity (MFI) of hASC-M, hASC-T, hASC-TS and hASC-TE cell populations was reported on the x-axis. Black line curves represent isotypic control; gray curves represent fluorescence peaks.

During culture, hASCs phenotypic characterization was conducted to establish whether transduction with *hTERT* and co-transduction with either *hTERT* + *SV40* or *hTERT* + *E6/E7* could modify the expression of the original mesenchymal marker profiles as well as markers involved in cell homing and stemness. For this reason, the expression of CD90, CD73, CD105, CD34, CD45, HLA-I and HLA-II, which have been described as MSC immunophenotypes was evaluated on all cell lines with fluorescence-activated cell sorting (FACS) analysis [21]. We found that the original hASC-M cultures and all transduced cell populations were positive for CD90, CD73, CD105 and HLA-I, and negative for CD34, CD45 and HLA-II, thus confirming the MSC immunophenotype (Table 3). Of note, CD105 showed a decrease in hASCs-TS and hASCs-TE cell surface expression (48.1% and 37.0%, respectively) compared to hASCs-M and hASCs-T, but all cell populations displayed a similar membrane distribution (Figure 5).

**Table 3:** Relative expression percentages of surface markers analyzed by flow cytometry

	**hASC-M**	**hASC-T**	**hASC-TS**	**hASC-TE**
**CD90**	81.0 ± 3.4	70.0 ± 17.0	96.3 ± 1.6	77.0 ± 21.0
**CD73**	94.0 ± 2.4	96.0 ± 2.1	85.0 ± 2.3	92.5 ± 3.6
**CD105**	83.0 ± 5.1	81.0 ± 0.3	48.1 ± 2.0	37.0 ± 16.0
**CD34**	1.5 ± 0.1	1.2 ± 0.5	0.6 ± 0.1	0.9 ± 0.3
**CD45**	0.8 ± 0.3	1.2 ± 0.1	1.0 ± 0.2	0.5 ± 0.1
**HLA-I**	96.3 ± 2.1	80.0 ± 8.0	98.0 ± 0.4	92.0 ± 6.0
**HLA-II**	0.6 ± 0.2	0.8 ± 0.4	1.6 ± 0.6	0.9 ± 0.3
**CD44**	97.0 ± 0.4	94.0 ± 3.0	98.7 ± 0.2	99.5 ± 0.3
**CD106**	12.0 ± 6.0	30.5 ± 12.0	12.0 ± 0.2	46.8 ± 19.8
**CD146**	4.8 ± 1.0	4.1 ± 0.4	26.0 ± 6.0	60.0 ± 14.0
**SSEA-4**	23.0 ± 2.8	29.0 ± 3.0	16.0 ± 6.0	30.0 ± 12.0

Phenotypic panel of markers analyzed by flow cytometry on hASC-M, hASC-T, hASC-TS and hASC-TE cells. Relative expression percentages of each antigen are reported. Values are the mean ± S.E. of five independent experiments.

We further investigated the expression of CD44 (H-CAM) and CD106 (VCAM-1) as homing markers [22] and CD146 (MUC18), and SSEA-4 (Stage-Specific Embryonic Antigen-4) as stemness markers [23,24]. All hASC cultures showed 100.0% positivity for the homing cell marker CD44, before and after transduction. To the contrary, the percentage CD106 positive cells significantly increased up to 46.8% in hASC-TE (Table 3) and showed a more homogeneous antigen membrane distribution compared to the other cell populations (Figure 5).

Interestingly, the CD146 surface expression was increased in hASC-TS and hASC-TE cells (26.0% and 60.0%, respectively) compared to hASC-M and hASC-T cells, while the embryonic marker SSEA-4 was equally expressed in all cell populations.

### Immortalized hASC-TS but not hASC-TE significantly reduced their differentiation potential

**Figure 6:**
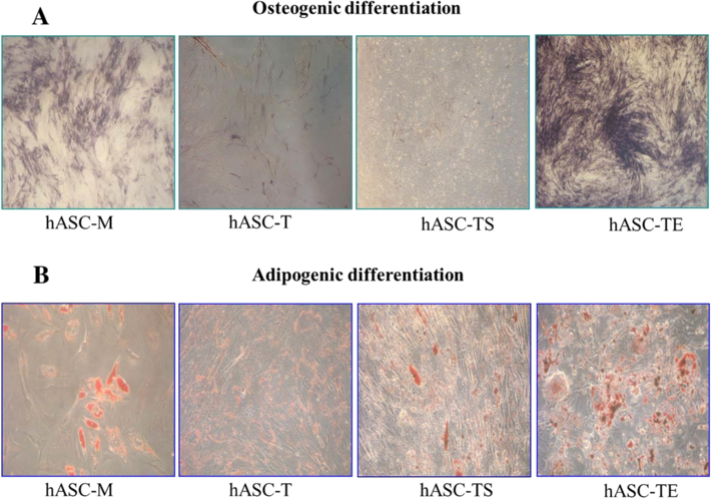
**Differentiation potential of hASC-M, hASC-T, hASC-TS and hASC-TE cells.** hASC-M (P8), hASC-T (P8), hASC-TS (P25) and hASC-TE (P25) cells were cultured under osteogenic and adipogenic differentiation conditions. **A**: Osteogenic induction was evaluated by alkaline phosphatase activity after 14 days of culture. **B**: Assessment of adipogenic differentiation by Oil Red O staining after 21 days of culture is shown. Magnification 10X.

We further investigated the capability of immortalized hASC-TS and hASC-TE cell lines to retain differentiation potential. hASC-M (P8), hASC-T (P8), hASC-TS (P25) and hASC-TE (P25) cells were cultured under osteogenic and adipogenic differentiation conditions. As shown in Figure 6A, hASC-M cells were able to differentiate into the osteogenic lineage after 14 days of induction. Similarly, hASCs-T, although less intense than control, showed a positive staining. Finally, we found that hASC-TS cells lost their differentiation potential, while all the hASC-TE cell lines retained a significant capability to differentiate into the osteogenic cell lineage.

Adipogenic induction evidenced that hASC-M and, to a lesser extent, hASC-T and hASC-TS cells stained for Oil Red O after 21 days. Conversely, hASCs-TE adipogenic potential was similar to non-transduced cells (Figure 6B).

### Immortalization of hASCs did not substantially reduce their angiogenic potential

It is widely demonstrated that hASCs are able to induce angiogenesis through the production and secretion of angiogenic factors and cytokines [25-27], such as HGF and VEGF [28,29]. So, we investigated whether the immortalized hASCs-TS and hASCs-TE continue to produce these growth factors *in vitro*. For this aim, conditioned media (CM) from hASCs-M and hASCs-T (passages 5-13-21), hASCs-TS and hASCs-TE (passages 16-22-38) were collected and, subsequently, HGF and VEGF concentration was measured. The values were normalized by the total number of cells and expressed as pg/10^5^ cells.

**Figure 7:**
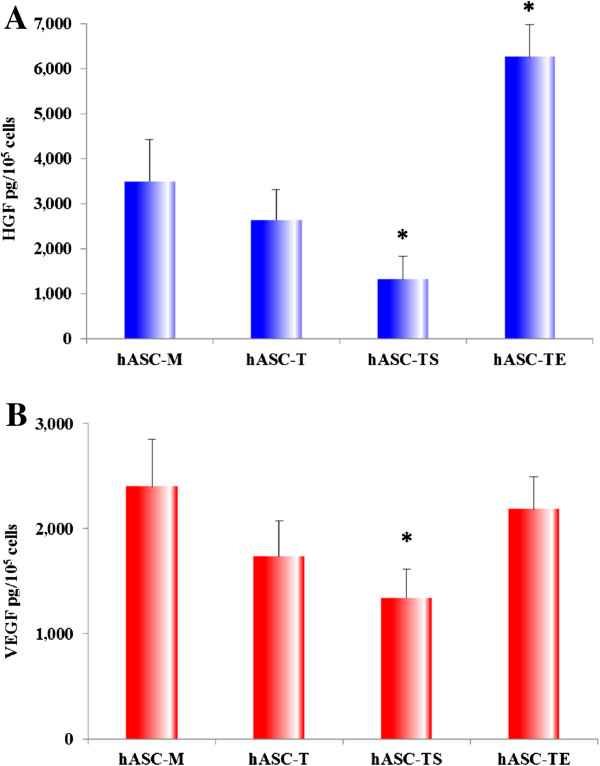
**Angiogenic potential of immortalized hASCs.** Conditioned media from hASCs-M and hASCs-T (passages 5-13-21), hASCs-TS and hASCs-TE (passages 16-22-38) were collected and subsequently analyzed by ELISA assay to evaluate the concentration of HGF and VEGF. For each sample analyzed, HGF and VEGF secretion were normalized by the total number of cells and expressed as pg/10^5^ cells. **A)** The HGF secretion was significantly decreased in hASC-TS cell lines and increased in hASC-TE cell lines. **B)** The VEGF secretion was similar in hASC-M and hASC-TE cell lines but significantly decreased in hASC-TS cell lines. Each histogram represents the mean ± SD of four different experiments. *P <0.05 vs hASC-M protein levels.

As shown in Figure 7A, hASCs-M produced about 3,500 pg/10^5^ cells of HGF in CM after 72 hours of culture, thus confirming that this growth factor is highly produced by primary cultures of hASCs. The level of HGF secreted by hASC-T cells were slightly reduced compared to the parental hASCs-M. On the other hand, hASC-TS cell lines showed a statistically significant (*P* <0.05) reduction of HGF secretion compared to the parental hASCs-M, but a significant increased level was secreted by hASC-TE cell lines (*P* <0.05).

The amount of VEGF secreted by hASCs-M cell lines in CM (Figure 7B) was about 2,500 pg/10^5^ cells. As observed for HGF production, hASC-T cell lines showed reduced levels of VEGF in CMs. Likewise, a statistically significant (*P* <0.05) reduction of VEGF was observed in all hASCs-TS CMs compared to the parental hASCs-M. To the contrary, a significant amount of VEGF was detected in hASCs-TE CM similar to the levels secreted by the parental hASCs-M.

### Immortalized hASC-TS and hASC-TE showed significant changes in karyotype and *in vitro* transformation

**Figure 8:**
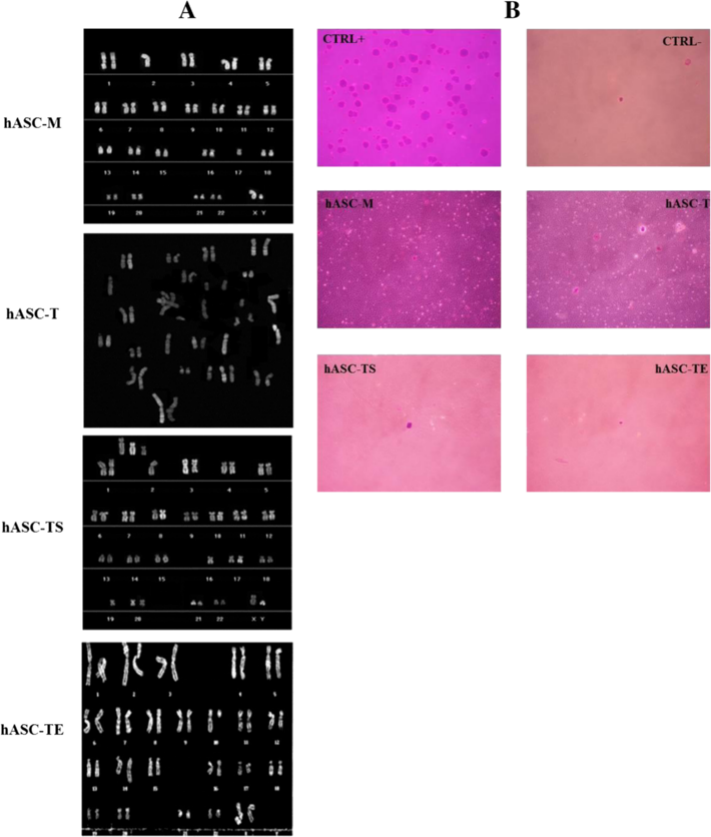
**Evaluation of chromosomal modifications and *in vitro *transformation. A**: Representative karyotype analysis of hASC-M, hASC-T, hASC-TS and hASC-TE cells at culture passage 26 evidencing alterations in all transduced-cells. **B**: Soft agar assay of hASCs. Only positive control gave rise to colony formation. Positive control: PC-3 cell line (tumor prostate); negative control: medium. Magnification 10X.

To assess whether transduction of hASCs cells either with hTERT alone or in combination with SV40 and HPV E6/E7 could induce chromosomal modifications, cytogenetic studies were conducted. Representative examples of hASC-M, hASC-T, hASC-TS and hASC-TE karyotype analysis at culture passage 26 are reported in Figure 8A. None of the hASC-M cell lines analyzed showed chromosomal alterations, whereas chromosomal aberrations and unbalanced translocations were evidenced in hASC-T, hASC-TS and hASC-TE cells. In particular, hASC immortalization with *hTERT* and *SV40* determined a significant alteration of the normal cell karyotype, producing cells with three additional chromosomes. To further understand how this altered karyotype could affect cell behavior and, more specifically, neoplastic transformation, a soft agar assay was performed. As shown in Figure 8B, although elevated cell number and prolonged culture time (21 or 28 days) were used as experimental conditions, only positive control (PC-3, prostate tumor) formed colonies. All cell lines behaved like the negative control (medium only).

### hASC-TS and hASC-TE cells can be successfully transduced with GFP for further *in vivo* tracking and homing studies

**Figure 9:**
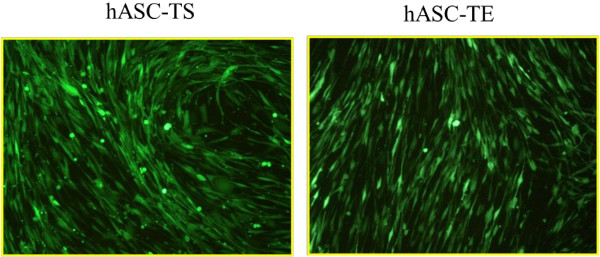
**Fluorescence microscopy images of hASC-TS and hASC-TE fluorescent cells.** hASC-TS and hASC-TE cells were transduced with GFP-lentiviral vectors. Magnification 10X. GFP, green fluorescent protein.

Due to the importance of producing labeled hMSC lines for investigating their homing and tracking *in vivo*[30], we also investigated the possibility of stably labeling hASCs-TS and hASCs-TE with GFP. For this aim, hASC-TS and hASC-TE cell lines were transduced with lentiviral particles containing the *GFP* gene. As shown in Figure 9, the hASC-TS and hASC-TE transduced lines expressed GFP protein, albeit with different transduction efficiency. In fact, hASC-TS cells appeared brighter than the hASCs-TE. Furthermore, GFP-fluorescent-labeled cells were able to proliferate continuously, maintaining their GFP expression and increasing their cell number (data not shown).

## Discussion

Human adipose tissue may represent an interesting reservoir of cells for therapeutic approaches. However, since the hASC population is not characterized by prolonged lifespan and stable characteristics, these aspects still pose significant limitations to their clinical use [31-33]. Different immunophenotypic and functional criteria have been proposed to better characterize hASCs [34], nevertheless the problem remains partially unsolved.

The cell immortalization process could be performed on primary hASC culture preparations in order to bypass the problems related to limited lifespan and cell heterogeneity [35,36]. It is well known that hASCs release significant amounts of cytokines and growth factors [37], and cell immortalization may be useful in facilitating the production/purification of secreted factors by bypassing limits imposed by the occurrence of senescence (scale-up process).

Considering these reasons, in the present work we immortalized four different hASCs primary cultures. All the immortalized lines were phenotypically and functionally characterized and compared to native non-transduced hASCs. Immortalized cells’ capability to release the angiogenic factors HGF and VEGF was also evaluated together with their ability to express GFP in a stable manner in order to potentially use these cells for *in vivo* cell-tracking.

Two different cell immortalization approaches were used. We started using the *hTERT* gene to avoid use of viral proto-oncogenes. Moreover, the successful immortalization of adipose tissue stromal cells has been previously described [38-40]. In our studies we did not achieve the expected results, despite the use of different MOI (Multiplicity of Infections) values and antibiotic-selection. Further attempts were done by treating *hTERT*-transduced cells with Y-27632 (data not shown), a Rho kinase inhibitor which has been demonstrated to efficiently immortalize human keratinocytes [41], but this experimental procedure also failed. Additionally, hASC-T cells showed elevated telomerase activity compared to non-transduced cells, but were unable to proliferate and expand *in vitro* (data not shown).

Thus, we decided to combine *hTERT* with *SV40* or *HPV E6/E7* genes, either by *ex-novo* transduced cells or with previously *hTERT*-transduced cells. In all cases we obtained cells with a prolonged *in vitro* proliferation rate and elevated cell expansion capabilities. PDL values showed high growth rates of both hASC-TS and hASC-TE cell lines. To the contrary, hASCs-M and hASCs-T were totally unable to grow after 50 to 80 days of culture. Higher PDL values were obtained by hASCs-TS compared to hASCs-TE. This could be linked in part to a lower doubling time required by hASCs-TS, although other mechanisms may determine this difference. For example, it is well established that viral *SV40* and *HPV E6/E7* genes differently affect cell-cycle check-points [42]. Additional important differences between immortalized hASCs-TS and hASCs-TE, and control hASCs-M and hASCs-T were detected. Indeed, it was observed that hASCs-TS and hASCs-TE retained a “fibroblast-like” cell morphology and did not express the β-galactosidase senescence marker over time. Conversely, the control hASCs-M and hASCs-T enlarged their size in culture and showed β-galactosidase activity. At least in part, these data concord with what was observed for proliferation; in fact, senescent cells lose their ability to divide although they tend to remain viable for a long time.

Immunophenotypic analysis revealed that immortalization did not affect cell surface marker expression substantially compared to non-transduced cells and most importantly, the expression remained unaltered over time. The only exceptions were the increase of CD106 in hASC-TE and CD146 in hASC-TS and hASC-TE cells when compared to non-transduced cells. The reason and significance of these variations still need to be clarified.

Our study showed that hASC-TS and hASC-TE cells differed in terms of differentiation capabilities. hASC-TS lines were able to differentiate only into adipogenic-like cells, while hASC-TE retained the capacity to differentiate into adipogenic and osteogenic cell lineages. Our results confirm previous data on the differentiation potential of immortalized cells. In fact, it has been demonstrated that co-expression of *hTERT* and *HPV-E7* in human pre-adipocytes did not alter their ability to differentiate [15]. In addition, Tátrai *et al*. [43] recently reported that hASCs immortalized by co-transducing cells with *hTERT* and *SV40* were unable to differentiate into an osteogenic lineage. The same authors evidenced that hASC^SV40+hTERT^ cells showed an aggressive *in vitro* behavior but were unable to form tumors in NOD/SCID gamma mice. Here, by using a soft agar *in vitro* transformation assay, we demonstrated that hASCs-TS and hASCs-TE were unable to give rise to colonies, albeit all immortalized lines showed significant chromosomal alterations. It remains to be investigated whether hASCs-TS and hASCs-TE are able to form tumors *in vivo*.

Several findings have demonstrated that hASCs are able to induce angiogenesis through a paracrine mechanism that, at least in part, could involve the secretion of HGF and VEFG [44,45]. Interestingly, it has recently been reported that rejuvenation or immortalization of adipose stromal cells may maximize their paracrine properties and improve their therapeutic potential. Madonna *et al*. [46] showed that overexpression of *hTERT* and *myocardin* genes in ASCs induced cell rejuvenation, increased their VEGF production and promoted revascularization and tissue repair when transplanted in a murine model of hindlimb ischemia. Recent studies have elucidated a possible role of CM with regenerative activities [47,48]. Song *at al*. [49] reported that transduction of MYC-immortalized hASCs with the constitutive active *(CA)-AKT* gene increased their VEGF secretion. Furthermore, injection of (CA)-AKT/MYC hASCs-CM promoted wound healing *in vivo*.

Here we demonstrated, for the first time, that hASCs immortalization with either *hTERT* + *SV40* or *hTERT* + *E6/E7* did not substantially affect their potent angiogenic capabilities. Indeed, although hASCs-TS showed a decrease in HGF and VEGF secretion compared to hASCs-M CM, the total amount of secreted factors remained elevated. More interesting, hASCs-TE showed an increased production of HGF and a similar VEGF secretion compared to hASCs-M CM.

Both hASC-TS and hASC-TE cell lines retained HGF and VEGF production over time. Further *in vivo* studies will need to clarify whether the different secretion level obtained from hASC-TS and hASC-TE CM may differently affect their angiogenic potential. Overall, these data suggest that hASCs-TS and hASCs-TE CM may be useful for therapeutic strategies.

An important aspect to consider when proto-oncogenes and lentiviral particles are used to immortalize cells is CM safety. In-depth *in vitro* and *in vivo* studies will be mandatory to evaluate the absence of potentially dangerous agents. Although onco-proteins, unlike oncogenes, cannot be replicated or amplified, thus reducing the risk of tumorigenesis, a comprehensive analysis (that is, proteomic studies) of CM will be needed to exclude the presence of altered proteins or factors. A recent study by Chen *et al*. [50] demonstrated that MYC-mediated hESC-MSCs immortalization was effective in producing an infinite supply of cells for the production of exosomes in the milligram range as either therapeutic agents or delivery vehicles. Interestingly, MYC protein was present in the transformed cells but was not detectable in either the CM or exosomes.

Concerning the lentiviral particles employment, it has to be considered that here we propose the administration of CM for therapeutic purposes and this may mitigate the risk from the integration of lentiviruses. Additionally, the use of the latest generation of lentiviral vectors with improved safety and efficacy, will further contribute to reduce risks [51,52].

Finally, in this study we showed that hASC-TS and hASC-TE can be stably transduced with GFP, allowing us to obtain fluorescent cells that maintained their proliferative activity. Preliminary results from our group indicate the possibility of obtaining clones of fluorescent-immortalized cells (data not shown); this may represent an important technical achievement that could lead to the use of GFP-immortalized cells for *in vivo* cell-tracking studies [53,54].

## Conclusion

Our study demonstrated that immortalized cell lines substantially maintained most of the original mesenchymal features and, in particular, the capability to produce significant amounts of angiogenic factors. Further investigations are needed to find other factors produced from these cells. However, by combining hASCs immortalization and their paracrine characteristics, we developed a “hybridoma-like model” that may have a potential application for discovering and producing molecules to use in regenerative medicine (scale-up process).

In fact, these cells’ growth rate and expansion together with their secretory capabilities over time could represent a new strategy for producing large amounts of soluble factors. Moreover, it is conceivable that conditioned media could be produced and used instead of the stem cells, thus reducing the potential risks connected to cell transplantation, particularly when heterologous cells are used. In addition, fluorescent-immortalized cells could be employed in *in vivo* cell-tracking experiments expanding their potential use in laboratory practice.

## Abbreviations

CM: Conditioned media; DT: Doubling time; FC: Flow cytometry; FITC: Fluoresceine isothiocyanate; GFP: Green fluorescent protein; hASCs: Human adipose-derived stromal cells; HGF: Hepatocyte growth factor; HPV-16: Human papilloma virus 16; hTERT: Human telomerase reverse transcriptase; MSCs: Mesenchymal stromal cells; PBS: Phosphate-buffered saline; PDL: Population doubling levels; PE: Phycoerythrin; SSEA-4: Stage specific embryonic antigen-4; VEGF: Vascular endothelial growth factor.

## Competing interests

The purpose of the present paper is to advance the field in an impartial manner. However, to ensure transparency, the authors acknowledge the following relationship: LB, AB, MS and AS are employees of Medestea Research and Production company. Although currently there are neither pending patents nor commercial developments worldwide, the company has long been involved in research projects aimed at the use of stem cells and their derivatives.

## Authors’ contributions

LB contributed to manuscript writing, mesenchymal stem cell isolation, expansion and immortalization, and GFP-transduction. AB contributed to phenotypic characterization, ELISA assays and data collection. MS was involved in mesenchymal stem cell isolation, expansion, immortalization and *in vitro* transformation and data collection. AS contributed to conception and design, and manuscript drafting and revision. AP took part in manuscript revision, and data analysis and interpretation. ABO and VC contributed to data collection and analysis. MD was involved in karyotype data collection and analysis. VT and VC contributed to differentiation experiments, and data collection and analysis. MLF was involved in GFP-lentiviral particles isolation and cell-transduction. EAP contributed to the study design and manuscript revision. GA was involved in the conception and design of the study, manuscript writing, and data analysis and interpretation. All authors read and approved the final manuscript.
